# Prognostic impact of individual resection and dissection margins in resected perihilar cholangiocarcinoma: retrospective study

**DOI:** 10.1093/bjsopen/zraf160

**Published:** 2026-02-03

**Authors:** Britte H E A ten Haaft, Hasan Ahmad Al-Saffar, Eva Roos, Mahsoem Ali, Heinz-Josef Klümpen, Lynn Nooijen, Lotte Franken, Geert Kazemier, Carlos Fernandez Moro, Joanne Verheij, Joris I Erdmann, Christian Sturesson

**Affiliations:** Department of Surgery, Cancer Center Amsterdam, Amsterdam UMC, University of Amsterdam, Amsterdam, the Netherlands; Division of Surgery and Oncology, Department of Clinical Science, Intervention and Technology, Karolinska Institute and Karolinska University Hospital, Stockholm, Sweden; Department of Digestive Diseases, Transplantation and General Surgery, Centre for Cancer and Organ Diseases, Copenhagen University Hospital, Rigshospitalet, Denmark; Department of Pathology, Cancer Center Amsterdam, Amsterdam UMC, University of Amsterdam, Amsterdam, the Netherlands; Department of Surgery, Cancer Center Amsterdam, Amsterdam UMC, University of Amsterdam, Amsterdam, the Netherlands; Department of Medical Oncology, Cancer Center Amsterdam, Amsterdam UMC, University of Amsterdam, Amsterdam, the Netherlands; Department of Surgery, Cancer Center Amsterdam, Amsterdam UMC, Vrije Universiteit, Amsterdam, the Netherlands; Department of Surgery, Cancer Center Amsterdam, Amsterdam UMC, Vrije Universiteit, Amsterdam, the Netherlands; Department of Surgery, Cancer Center Amsterdam, Amsterdam UMC, Vrije Universiteit, Amsterdam, the Netherlands; Department of Clinical Pathology and Cancer Diagnostics, Karolinska University Hospital, Stockholm, Sweden; Department of Pathology, Cancer Center Amsterdam, Amsterdam UMC, University of Amsterdam, Amsterdam, the Netherlands; Department of Surgery, Cancer Center Amsterdam, Amsterdam UMC, University of Amsterdam, Amsterdam, the Netherlands; Division of Surgery and Oncology, Department of Clinical Science, Intervention and Technology, Karolinska Institute and Karolinska University Hospital, Stockholm, Sweden

**Keywords:** radicality, liver surgery, planes, recurrence-free survival, overall survival

## Abstract

**Background:**

Various studies have reported on the prognostic impact of ductal margin and radial margin status in resected perihilar cholangiocarcinoma (PCCA). No study has considered differences in the prognostic impact of individual resection margins. This study investigated the prognostic impact of individual planes on survival.

**Methods:**

All patients undergoing surgery for PCCA at Amsterdam UMC and Karolinska University Hospital between January 2010 and May 2023 were included. Clinicopathological data were retrospectively retrieved. The primary outcomes were the prognostic significance of residual disease (< 1 mm to the nearest tumour growth) in individual dissection planes and resection margins for overall survival (OS) and disease-free survival (DFS), expressed as adjusted hazard ratios (aHRs).

**Results:**

Of 199 patients, 81 (41%) underwent radical resection and 118 (59%) were reported to have microscopic residual disease. Only a positive proximal bile duct resection margin was significantly associated with shorter OS (adjusted median OS 24 *versus* 36 months; aHR 1.64; 95% confidence interval (c.i.) 1.05 to 2.56; *P* = 0.031) and DFS (aHR 2.01; 95% c.i. 1.30 to 3.10; *P* = 0.002). Other positive resection margins and dissection planes did not carry any prognostic information for OS (*P*_interaction_ = 0.95) or DFS (*P*_interaction_ = 0.56). Similar results were obtained in a 90-day landmark sensitivity analysis.

**Conclusion:**

This study found that only tumour infiltration of the proximal bile duct resection margin was associated with worse prognosis, most likely reflecting the malignant behaviour of the disease rather than surgical failure. Larger prospective studies are needed to clarify the true prognostic impact of residual disease in individual resection planes to allocate patients to specific chemotherapeutic (neo)adjuvant treatments.

## Teaser

This multicentre retrospective study found that only tumour infiltration of the proximal bile duct resection margin was associated with worse prognosis in resected perihilar cholangiocarcinoma, most likely reflecting by poor tumour biology rather than surgical failure. Larger prospective studies are needed to clarify the true prognostic impact of residual disease in individual resection planes to allocate patients to specific chemotherapeutic (neo)adjuvant treatments.

## Introduction

Perihilar cholangiocarcinoma (PCCA) is a rare tumour that originates from the biliary tract^1^. Due to presentation at an advanced stage, PCCA is associated with a poor prognosis, with a 5-year survival rate below 10%^1^. Although surgical treatment provides the only chance for long-term survival, the 5-year survival rate after resection remains low (30%). Most patients (55–80%) experience disease recurrence within 2 years after surgery^2,3^.

Surgical treatment typically involves extrahepatic bile duct (EHBD) resection with or without hemihepatectomy^2^. For years, residual disease (R1) has been considered a highly relevant negative prognostic factor for survival and disease recurrence after resection^4^. Surgeons and pathologists make considerable efforts to achieve radical resections and identify all individual resection margins and dissection planes. In the recent literature, six standard resection margins and dissection planes have been postulated: the distal bile duct (DBD; common bile duct), proximal bile ducts (PBD; hepatic bile ducts), hepatic artery, portal vein, liver parenchyma resection, and periductal dissection planes^5^. In the absence of published evidence for PCCA and given the similarities between biliary and pancreatic duct cancers, adopting a similar approach to defining R1 resection, defined as cancer cells < 1 mm from any transection margin or dissection plane, is considered appropriate, as reported in the 2018 guidelines from the International Collaboration on Cancer Reporting (ICCR)^6^. Differences in the prognostic impact of, for example, ductal (DM), radial (RM), or individual margins were not considered^6–8^.

Traditionally, not all planes have been assessed, and there are various definitions for margin evaluation. Some Western series only reported DM, comprising both DBD and PBD margins^5^. Other series have reported longitudinal margins (LM), defined as DM plus liver parenchyma margin^5,9–11^. Because of its negative association with overall survival (OS), recent Eastern and Western studies have highlighted the value of reporting RM, which includes the hepatic artery, portal vein, and periductal margin^5,9–11^. However, no study has assessed the prognostic value of individual resection margins and dissection planes. With emerging systemic treatments for PCCA, understanding the true relationship between residual disease and OS or disease-free survival (DFS) is critical. Thus, the aim of the present study was to investigate the association between individual resection margins and dissection planes and survival.

## Methods

### Study design and participants

Pathology reports of adults with confirmed PCCA who underwent hemihepatectomy including EHBD resection at the Amsterdam University Medical Center (UMC) in the Netherlands, and at Karolinska University Hospital in Sweden between 1 January 2010 and 31 May 2023 were included in the study. Patients with only intraductal papillary neoplasms of the bile duct, benign disease, and other biliary tract carcinomas involving the hilum were excluded. Patients who underwent liver transplantation or solely EHBD resection were also excluded.

The requirement for informed consent was waived by the Medical Ethics Review Committee of the Amsterdam UMC (#2024.0635) and the Regional Ethics Board in Stockholm (2022-06962-02).

### Data assessment

Data were retrospectively retrieved from original pathology reports from the local electronic patient records. At Amsterdam UMC, these reports were evaluated by three independent investigators (B.T.H., E.R., and L.F.). If a pathology report was incomplete or unclear, additional assessment of the specimen was conducted by hepatopancreatobiliary (HPB) pathologists (J.V. and E.R.). At Karolinska University Hospital, all data were evaluated by one independent investigator (H.A.), with reports supplied by HPB pathologists.

### Perioperative work-up and treatment

At both Amsterdam UMC and Karolinska University Hospital, patients were evaluated for resectability during the local multidisciplinary team meeting. All patients with suspected PCCA were discussed, with the participation of at least two liver surgeons, an abdominal radiologist, a medical oncologist, and a pathologist. Resectability was defined as the possibility of a radical biliary and liver parenchyma resection, with or without portal venous resection and anastomosis. Encasement of the future liver remnant artery or vein, extrahepatic disease, or lymph node metastasis distal to the hepatoduodenal ligament was generally considered a contraindication to resection. Severe cirrhosis (Child–Pugh B/C) was considered a contraindication to resection. Any patient with a liver function of < 2.7%/min/m^2^ at scintigraphy (Amsterdam UMC) or a radiological future liver remnant volume of < 30% of standardized total liver volume (Karolinska University Hospital)^11^ was referred for portal vein embolization (PVE). Resection type was based on the Bismuth–Corlette (BMC) classification and vascular involvement of the future liver remnant. Surgical resections included conventional or extended left/right hemihepatectomy, all with EHBD. Routine lymphadenectomy comprised the hepatoduodenal ligament (stations 12a, 12b, 12p) and hepatic artery (station 8a) lymph nodes. In the case of macroscopically suspected metastasis, the retropancreatic (station 13), coeliac (station 9), and aortocaval (station 16b) lymph nodes were sampled. Frozen sections were performed at the surgeon’s discretion. At that time, ductal re-resection was considered in the case of positive frozen sections, and pancreatoduodenectomy (PD) was performed if tumour invasion extended into the pancreatic head. At Amsterdam UMC, postoperative regimens included adjuvant capecitabine or gemcitabine plus cisplatin from September 2015 to December 2022 as part of the ACTICCA trial^(12)^. Some patients with locally advanced PCCA received induction chemotherapy as part of the IMPACCA trial^13^. Induction chemotherapy at Karolinska University Hospital was only considered in select patients who were reassessed at multidisciplinary team meetings in case of a significant response to palliative chemotherapy. At Karolinska University Hospital, all patients were offered adjuvant gemcitabine until 2018, after which single capecitabine was opted for as standard treatment following the BILCAP trial in 2019^14^.

### Pathology assessment

After surgery, specimens were assessed by pathology departments. The evaluation was performed by expert pathologists, who followed the *Eighth Edition AJCC Cancer Staging Manual*^15^ and ICCR guidelines^6^. Prior to assessment, specimens were stained and fixed in 10% formalin. For a complete overview of the pathology assessment, see *supplementary methods*^6,16^.

**Fig. 1:**
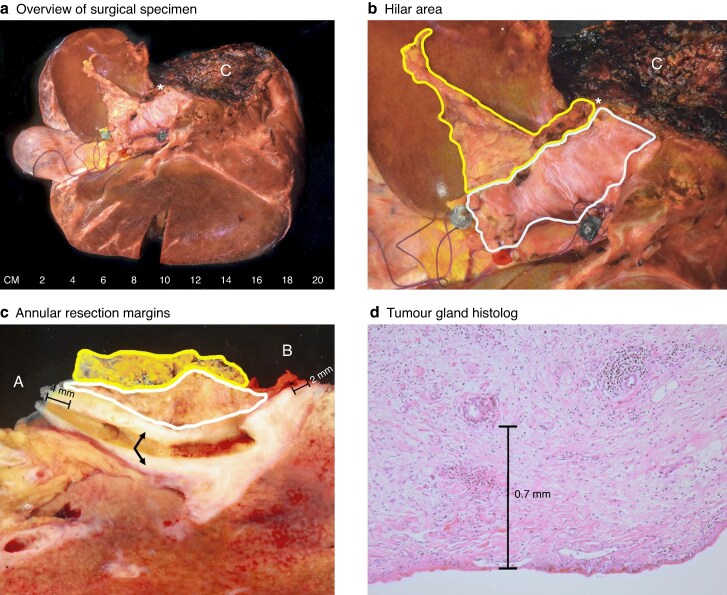
Images of the surgical specimen **a** Overview of the surgical specimen in extended right hemihepatectomy (including segment 1) with extrahepatic bile duct resection. **b** Close-up image of the hilar area. The fine fibres of the smooth peritoneal surface can be appreciated (outlined in white), as well as the slightly irregular periductal dissection plane (outlined in yellow). The green bead indicates the common bile duct, the blue bead indicates the portal vein, and the red bead indicates the hepatic artery. The left hepatic duct is indicated by the asterisk. **c** The annular resection margins of the common bile duct (A) and left hepatic duct (B) were sampled, and the underlying tissue was coloured using green and red ink, respectively. The specimen was cut along the bile duct after probing. Part of the bile duct shows a white, fibrotic, and thickened wall due to tumour involvement (arrows), with a 4-mm clearance from the common bile duct margin and a 2-mm clearance from the left hepatic duct. **d** Histological section showing a tumour gland in relation to the peritoneal surface. The periductal dissection plane was not involved in this specimen, as shown in **c**. C, liver parenchyma. Image and legend reused from Roos *et al*.^5^, licensed under CC BY 4.0 (http://creativecommons.org/licenses/by/4.0/).

No residual disease (R0) was defined as a tumour-free margin of ≥ 1 mm in all assessed margins and planes, whereas residual disease (R1) was defined as < 1 mm to the nearest tumour growth in any assessed margin during the final pathology assessment^6^. The evaluated resection margins and dissection planes included the PBD (hepatic bile ducts) resection margin, DBD (common bile duct) resection margin, hepatic artery resection margin, portal vein resection margin, liver parenchyma resection margin, and periductal (circumferential or hepatoduodenal ligament) dissection plane (*Fig. 1*).

### Outcomes and definitions

The primary outcome measure was the association between residual disease in individual resection margins or dissection planes and OS. The secondary outcome measure was the association between residual disease in individual resection margins or dissection planes and DFS. In addition, comparisons were performed between LM (DBD and PBD resection margin), RM (liver parenchyma, hepatic artery, portal vein resection margins, and periductal dissection plane), and LM + RM according to categories defined in previous studies^10,11^. OS was defined as the time from surgery to death or last follow-up. DFS was defined as the time from surgery to recurrence, death from any cause or last follow-up, whichever came first. EPRs were assessed for clinicopathological parameters. All parameters assessed are listed in the *supplementary methods*.

### Statistical analysis

Categorical variables are reported as frequencies and percentages. Continuous variables are reported as the median with interquartile range (i.q.r.). Baseline characteristics for all patients were stratified according to residual disease (R) status, and the significance of differences at baseline was determined using Pearson’s χ^2^ test for categorical data and the Mann–Whitney *U* test for continuous data. The median follow-up was estimated using the reverse Kaplan–Meier method.

Multivariable Cox regression models were used to assess the association of R status from different resection margins and dissection planes with OS and DFS, expressed as hazard ratios (HRs). These models included all resection margins and dissection planes and several potential confounders, including pre- and intraoperative factors. Continuous variables were modelled using restricted cubic splines, and the proportional hazards assumption was investigated using visual inspection of scaled Schoenfeld residuals and the Grambsch–Therneau test. Flexible parametric Royston–Parmar survival models followed by regression standardization was used to show the association of a positive R status with OS and DFS for each resection margin and dissection plane^17^. A sensitivity analysis with a 90-day landmark was performed to evaluate long-term OS while excluding postoperative mortality. Missing data were handled using flexible multiple imputation models that were congenial with the analysis model (50 imputed data sets; 30 burn-in iterations) under the missing-at-random assumption. A full explanation of the statistical analyses is presented in the *supplementary methods*.

In this study, a two-sided *P* < 0.05 was considered statistically significant. Statistical analyses were performed using R version 4.4.1 (R Foundation for Statistical Computing, Vienna, Austria) and Stata version 18 (StataCorp, College Station, TX, USA), using the rms package and the stpm3 module^18,19^.

## Results

### Patient characteristics

Baseline patient characteristics are presented in *Table 1*, with the number of missing values per variable provided in *Table S1*. In all, 199 patients with pathologically confirmed PCCA were included in the study; 81 patients (41%) were reported as R0 and 118 patients (59%) were reported as R1 (median age 64 and 67 years old, respectively). Most patients (186, 93%) were diagnosed as BMC type ≥ III. A PVE and an additional PD were performed more often in the R0 than R1 group (PVE, 27 *versus* 24 (*P* = 0.039); additional PD, 6 *versus* 1 (*P* = 0.014)), whereas postoperative mortality within 90 days was higher in the R1 than R0 group (16 *versus* 4%, respectively; *P* = 0.047).

**Table 1:** Baseline characteristics according to residual tumour classification

	R0 (*n* = 81)	R1 (*n* = 118)	P*
Age (years), median (i.q.r.)	64 (56–72)	67 (57–74)	0.194
**Sex**			0.781
Male	51 (63%)	72 (61%)	
Female	30 (37%)	46 (39%)	
BMI (kg/m^2^), median (i.q.r.)	24 (22–27)	24 (22–26)	0.493
**ASA grade**			0.094
I–II	69 (85%)	89 (75%)	
III	12 (15%)	29 (25%)	
**Bismuth–Corlette classification**			0.007
Type I–II	9 (11%)	4 (3%)	
Type IIIA	51 (63%)	58 (49%)	
Type IIIB	11 (14%)	24 (20%)	
Type IV	10 (12%)	32 (27%)	
Prior abdominal surgery‡	15 (31%)	20 (29%)	0.810
**Preoperative**			
Bilirubin (μmol/l), median (i.q.r.)	16 (10–28)	15 (9–25)	0.360
Albumin (g/l), median (i.q.r.)	34 (29–40)	34 (30–39)	0.900
CA19-9† (U/mL), median (i.q.r.)	105 (46–259)	173 (36–443)	0.477
Function (Amsterdam UMC) (%/min/m²), median (i.q.r.)	4 (3–5)	4 (3–6)	0.242
Volume (Karolinska) (%), median (i.q.r.)	35 (29–41)	34 (30–38)	0.496
Preoperative treatment	17 (22%)	28 (24%)	0.716
**Biliary drainage**			0.995
Percutaneous	16 (20%)	23 (20%)	
Endoscopic	37 (46%)	56 (48%)	
Both	13 (16%)	18 (15%)	
Portal vein embolization	27 (33%)	24 (20%)	0.039
**Type of resection**			0.499
Right hemihepatectomy	21 (26%)	27 (23%)	
Extended right hemihepatectomy	34 (42%)	41 (35%)	
Left hemihepatectomy	19 (24%)	34 (29%)	
Extended left hemihepatectomy	7 (9%)	16 (14%)	
Additional pancreatic resection	6 (7%)	1 (1%)	0.014
**Vascular reconstruction**	24 (30%)	33 (28%)	0.799
Arterial‡ (hepatic artery)	1 (1%)	1 (1%)	
Venous‡ (portal vein)	17 (21%)	18 (15%)	
Unknown	6 (7%)	14 (12%)	
**Postoperative complications**			
Clavien–Dindo grade ≥ III, < 90 days	50 (65%)	88 (77%)	0.080
Mortality, < 90 days	4 (5%)	16 (14%)	0.047
Adjuvant therapy	28 (36%)	23 (20%)	0.013
**Medical centre**			0.608
Amsterdam UMC (NL)	53 (65%)	73 (62%)	
Karolinska Hospital (SWE)	28 (35%)	45 (38%)	

Values are *n* (%) unless otherwise stated. †Last available outcome before surgery. ‡Only available for Amsterdam UMC. i.q.r., interquartile range; BMI, body mass index; ASA, American Society of Anesthesiologists; CA19-9, carbohydrate antigen 19-9; NL, Netherlands; SWE, Sweden. *Pearson’s χ2 test for categorical data and the Mann–Whitney U test for continuous data.

Tumour pathology characteristics according to R status are summarized in *Table 2*. Only differentiation grade was significantly worse in the R1 group (*P* = 0.029). Residual disease was reported most often in the periductal dissection plane (51%, 60 of 118) followed by the PBD resection margin (40%, 47 of 118), and the liver parenchyma resection margin (29%, 34 of 118). Pathology reports from Karolinska University Hospital were complete. In Amsterdam UMC, reports were available for 53 R0 and 73 R1 patients. In 24 of 53 patients in the R0 group and in 32 of 73 patients in the R1 group, one or more resection margins or dissection planes were missing (R0 group: DBD, 0 missing; PBD, 1; portal vein, 8; hepatic artery, 14; liver parenchyma, 4; periductal, 13; and R1 group: DBD, 2; PBD, 0; portal vein, 11; hepatic artery, 27; liver parenchyma, 7; periductal, 10). Additional data on frozen sections are reported in *Table S2*.

**Table 2:** Tumour pathology characteristics according to residual tumour classification

	R0 (*n* = 81)	R1 (*n* = 118)	P*
Tumour diameter (mm), median (i.q.r.)	33 (21–40)	28 (22–40)	0.350
**Tumour stage**: **T category**†			0.176
pT1	4 (5%)	2 (2%)	
pT2A	25 (31%)	22 (19%)	
pT2B	27 (34%)	44 (38%)	
pT3	18 (23%)	36 (31%)	
pT4	6 (8%)	11 (10%)	
**Tumour stage: N category**†			0.079
pN0	53 (65%)	62 (53%)	
pN1	26 (32%)	43 (37%)	
pN2	2 (3%)	11 (10%)	
**Tumour stage: M category**†			0.650
pM0	77 (96%)	112 (97%)	
pM1¶	3 (4%)	3 (3%)	
**Differentiation grade**			0.029
Poor	10 (14%)	32 (31%)	
Moderate	52 (71%)	63 (60%)	
Good	11 (15%)	10 (10%)	
Perineural invasion	63 (85%)	101 (86%)	0.677
Microscopic angioinvasion	31 (38%)	64 (55%)	0.069
Liver parenchyma invasion‡	28 (53%)	50 (69%)	0.202
**Residual disease**§			
Distal (common bile duct)	0 (0%)	16 (14%)	NE
Proximal (hepatic duct)	0 (0%)	47 (40%)	NE
Portal vein	0 (0%)	20 (17%)	NE
Hepatic artery	0 (0%)	5 (4%)	NE
Liver parenchyma	0 (0%)	34 (29%)	NE
Periductal/circumferential	0 (0%)	60 (51%)	NE
**Missing resection margins and dissection planes**‡			0.658
None (complete)	29 (55%)	41 (56%)	
1 missing	11 (21%)	17 (23%)	
2 missing	9 (17%)	10 (14%)	
3 missing	4 (8%)	3 (4%)	
4 missing	0 (0%)	2 (3%)	

Values are *n* (%) unless otherwise stated. †Following the *Eighth Edition AJCC Cancer Staging Manual*^15^. ‡Only available for Amsterdam UMC. §Following the 2018 guidelines from the International Collaboration on Cancer Reporting^6^. ¶Underwent surgery despite the presence of metastatic disease because of various clinical considerations (for example, young age). i.q.r., interquartile range; NE, not evaluated. *Pearson’s χ2 test for categorical data and the Mann–Whitney U test for continuous data.

After a median follow-up of 67 (i.q.r. 40–135) months, 100 patients (51%) were diagnosed with disease recurrence on imaging or after biopsy and 139 patients (71%) had died. Median OS in the overall cohort was 30 (95% c.i. 28 to 36) months, with 1-, 3-, and 5-year OS rates of 79% (95% c.i. 73 to 85), 43% (95% c.i. 36 to 51), and 30% (95% c.i. 23 to 38), respectively. There was no evidence that an R1 resection was associated with shorter OS than an R0 resection in both unadjusted analyses (median OS 28 (95% c.i. 23 to 35) *versus* 36 (95% c.i. 31 to 57) months, respectively; HR for R1 *versus* R0 1.22; 95% c.i. 0.86 to 1.69; *P* = 0.26) and adjusted analyses (median OS 33 (95% c.i. 27 to 41) *versus* 31 (95% c.i. 24 to 40) months, respectively; adjusted HR (aHR) 0.88; 95% c.i. 0.60 to 1.30; *P* = 0.50). Similar results were obtained in a sensitivity analysis with a landmark of 90 days. In that landmark analysis, the aHR for R1 was 0.89 (95% c.i. 0.60 to 1.32; *P* = 0.56). Similarly, there was no evidence for a difference in DFS between the R1 and R0 groups in both unadjusted analyses (median 17 (95% c.i. 12 to 20) *versus* 29 (95% c.i. 23 to 43) months, respectively; *P* = 0.079) and adjusted analyses (median 21 (95% c.i. 7 to 28) *versus* 22 (95% c.i. 16 to 29) months, respectively; *P* = 0.93). Compared with R0/N0, the HRs for the R0/N1-2, R1/N0, and R1/N1-2 groups were 1.43 (95% c.i. 0.83 to 2.46), 1.06 (95% c.i. 0.67 to 1.68), and 1.95 (95% c.i. 1.23 to 3.08), respectively (*Fig. S1*).

**Fig. 2:**
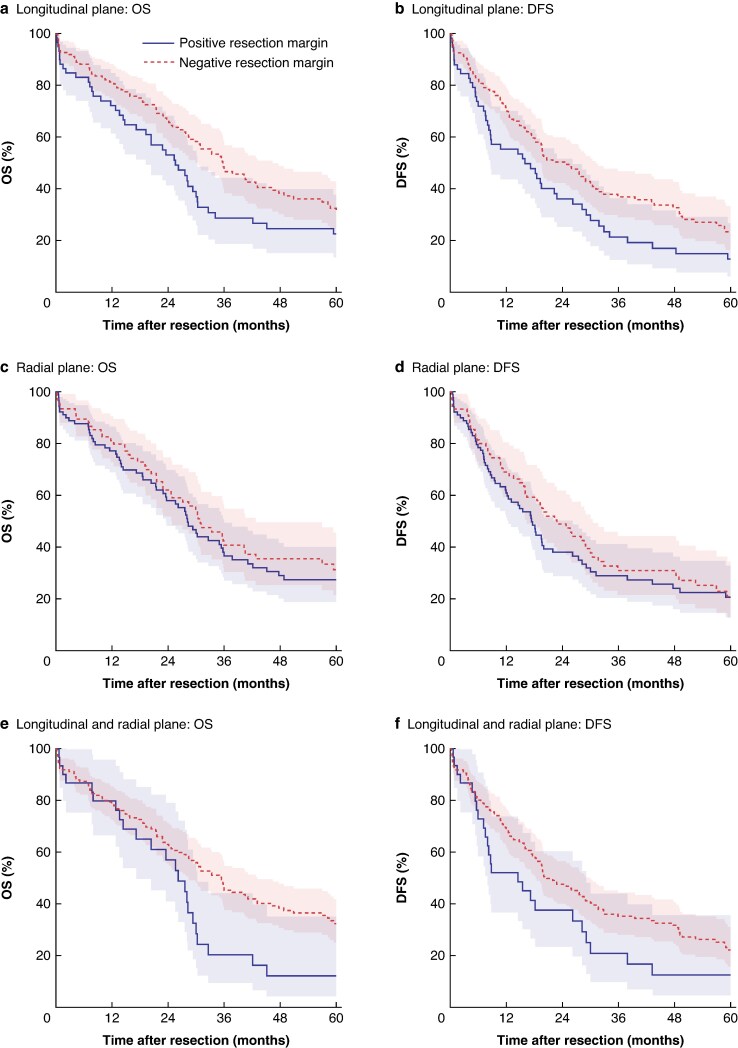
Kaplan–Meier curves of OS and DFS for longitudinal and radial resection planes **a** OS for longitudinal resection plane (HR 1.52; 95% c.i. 1.07, 2.16; *P* = 0.020). **b** DFS for longitudinal resection plane (HR 1.58; 95% c.i. 1.13, 2.22; *P* = 0.010). **c** OS for radial resection plane OS (HR 1.12; 95% c.i. 0.79, 1.58; *P* = 0.53). **d** DFS for radial resection plane (HR 1.18; 95% c.i. 0.85, 1.65; *P* = 0.330). **e** OS for longitudinal and radial resection plane (HR 1.49; 95% c.i. 0.97, 2.31; *P* = 0.068). **f** DFS for longitudinal and radial resection plane (HR 1.52; 95% c.i. 1.00, 2.31; *P* = 0.053). HRs were estimated using univariable Cox regression models, pooled across multiply imputed data sets. Shaded areas indicate the 95% c.i. OS, overall survival; DFS, disease-free survival; HR, hazard ratio; c.i., confidence interval.

The association of LM, RM, and LM + RM with OS and DFS is shown in *Fig. 2*. Notably, only a positive LM was associated with significantly shorter OS (median 26 *versus* 36 months; HR 1.52; 95% c.i. 1.07 to 2.16; *P* = 0.020) and shorter DFS (median 16 *versus* 25 months; HR 1.58; 95% c.i. 1.13 to 2.22; *P* = 0.010). In contrast, there was at most weak evidence for an association of positive RM and LM + RM with shorter OS, although confidence intervals were wide and compatible with both slightly longer OS and substantially shorter OS (*Fig. 2c–f*). There was no evidence that the association of LM with OS and DFS was different between patients with positive *versus* negative RM (*P*_interaction_ = 0.93 for OS; *P*_interaction_ = 0.81 for DFS).

**Fig. 3:**
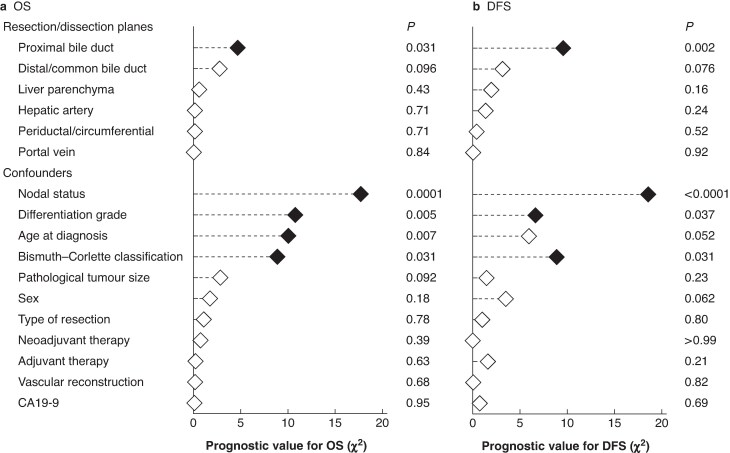
Prognostic value of resection margins and dissection planes in multivariable models **a** OS and **b** DFS. Variables with *P* < 0.05 are shown as closed diamonds. The χ^2^ values were derived from multivariable models including all variables listed in the figure and were pooled across multiply imputed data sets. Briefly, a higher χ^2^ indicates a stronger prognostic value of that variable for the outcome. OS, overall survival; DFS, disease-free survival.

The prognostic value of individual resection margins and dissection planes in multivariable models for OS and DFS is shown in *Fig. 3a*. Only a positive PBD resection margin was associated with significantly shorter OS (aHR 1.64; 95% c.i. 1.05 to 2.56; *P* = 0.031; *Fig. S2*) and DFS (aHR 2.01; 95% c.i. 1.30 to 3.10; *P* = 0.002; *Fig. S3*). Adjusted median OS was 36 months with a negative PBD resection (95% c.i. 30 to 44), compared with 24 months with a positive PBD resection margin (95% c.i. 17 to 33; adjusted difference 12 months (95% c.i. 1 to 23; *P* = 0.026)). Results were similar in a sensitivity analysis with a landmark of 90 days to account for the higher incidence of 90-day mortality in patients with R1 margins (4 *versus* 15%). In that landmark analysis, the aHR of positive PBD margin was 1.64 (95% c.i. 1.05 to 2.57; *P* = 0.029). A sensitivity analysis without multiple imputation for missing data and adjustment for confounders did not materially change the OS results (*Table S3*). A sensitivity analysis excluding the six patients with M1 disease did not materially affect the main outcome. In addition, results were not materially different in a post hoc analysis with additional adjustment for American Society of Anesthesiologists grade (I–II *versus* III–IV), PVE, and year of surgery.

Interaction analyses showed that the association of the PBD resection plane with OS was not moderated by pathological tumour size (*P*_interaction_ = 0.91) or differentiation grade (*P*_interaction_ = 0.88). Similarly, there was no evidence that the prognostic value of other resection margins or dissection planes was differed significantly between patients with a positive PBD resection margin and those with a negative PBD resection margin, both for OS (*P*_interaction_ = 0.95) and DFS (*P*_interaction_ = 0.56). In contrast, there was strong evidence that a positive PBD resection margin was especially associated with poorer survival in patients with N1 disease (HR 3.32; 95% c.i. 1.60 to 6.91) compared with patients with N0 disease (HR 1.51; 95% c.i. 0.83 to 2.75; *P*_interaction_ = 0.012; *Fig. S1*). The association between R1 margins and shorter OS was attenuated in patients who did not receive adjuvant therapy (HR 0.66; (95% c.i. 0.33 to 1.32) *versus* 1.49 (95% c.i. 0.99 to 2.24); *P*_interaction_ = 0.044).

## Discussion

This cohort study on the prognostic impact of residual disease of six individual resection margins and dissection planes on long-term outcomes found that only a positive PBD resection margin was associated with significantly shorter OS and DFS (aHR 1.64 (95% c.i. 1.05 to 2.56; *P* = 0.031) and aHR 2.01 (95% c.i. 1.30 to 3.10; *P* = 0.002), respectively). When comparing predefined subgroups (LM, RM, and LM + RM), only a positive LM was associated with significantly shorter OS and DFS.

This is the first study to have investigated all individual resection margins and dissection planes in PCCA. Instead of individual margins, previous studies have been performed on pooled margins: LM, RM, and LM + RM^9–11^. Remarkably, whereas the present study reported that only a positive LM was associated with significantly shorter OS and DFS, all other three studies^9–11^ reported that a positive RM was associated with a shorter OS and DFS. However, all three studies^9–11^ are limited by their single-centre study design and smaller sample size. Both studies^10,11^ also included patients from 2001 to 2019, potentially reflecting different pathological assessments compared with the present study (2010–23) and current practices. Notably, definitions of RM and LM differ highly between the studies, which may have contributed to different outcomes. In addition, in the present study, the adverse association between positive LM and OS or DFS was driven by the PBD resection margin, rather than the cumulative value of both the PBD and DBD resection planes. This highlights the importance of assessing individual rather than groups of margins or planes. Differences in outcomes may also be explained by the fact that previous studies did not exclude BMC type I patients who only underwent EHBD resection. Because the liver parenchyma resection plane is not present in the LM of BMC type I, its inclusion could have overestimated R0 in the overall assessment of the LM for all BMC types. In addition, R0 was universally defined as ≥ 1 mm according to the 2018 ICCR guideline^6^, which probably yielded higher R1 rates as opposed to other cohorts that used > 0 mm as the threshold for R0 as described by The College of American Pathologists in 2010^20–23^. Altogether, this may explain higher R1 rates (59%) in the present study population compared with previous cohorts^9–11,20,21,23^ from the same study period.

The adverse association between a positive PBD resection margin and OS may suggest the benefit of intraoperative longitudinal frozen section with further re-resection of positive PBD margins. However, no association between re-resection of the PBD and R1 was shown, probably due to the low number of patients (*Table 1*). Re-resection of the PBD is technically difficult without extending the liver resection (from conventional to extended left/right hemihepatectomy). In addition, extending the liver resection introduces increased risks for postoperative mortality. One may suggest the use of preoperative cholangioscopy to detect patients with a risk of positive PBD, and thus facilitate work-up for extended resections. However, several studies have shown no significant association between longer survival and re-resection after positive PBD frozen section^20,21^. In addition, the clinical value of intraoperative frozen sections of the PBD is questionable because of a relatively low sensitivity^21^. Although the present study has its limitations due to its retrospective nature, it questions the practice of achieving surgical success by intraoperative frozen margin assessment. In analogy of previous results on positive PBD frozen sections, a positive PBD resection margin, rather than an R1 status caused by other margins, may be the main indicator of poor tumour biology or bad anatomical localization (*Fig. 3* and *Fig. S1*). Thus, a positive PBD may serve as a surrogate for malignant behaviour with a highly invasive pattern and BMC type 4, rather than representing surgical failure necessitating extension of the resection. As such, a positive PBD margin could be managed by implementing adjuvant chemotherapeutic strategies rather than intraoperative re-resection. However, this hypothesis has not been formally proven, because this study does not provide molecular or histopathological markers correlating with tumour aggressiveness. Future studies incorporating molecular profiling and translational research would be valuable in further elucidating the association between resection margins and tumour biology. Moreover, in addition to resection margin status, other prognostic factors, such as lymph node involvement (*Fig. S1*) or (micro)metastases^22^, should also be considered when considering additional systemic treatment.

The present study found no significant evidence that overall R1 resection was associated with shorter OS or DFS, although both numerically favoured the R0 group. The lack of significance could be explained by the limited statistical power of the study. Patient selection may also have contributed to the findings, because the present study primarily included more severe BMC types (III and IV), whereas other studies have included BMC type I or II. In addition, patients in the R1 group more frequently had a poor differentiation grade (31 *versus* 14%; *P* = 0.029), numerically higher microscopic angioinvasion (55 *versus* 38%; *P* = 0.069), and numerically higher pN2 classification (10 *versus* 3%; *P* = 0.079). This may explain the poor 90-day mortality in the R1 group, because these patients usually require more extensive resections. In addition, more extensive disease could result from the time gap between imaging and surgery. However, at the centres included in the present study, imaging is repeated if it is older than 4–6 weeks before surgery to minimize this risk. Finally, current R1 and R0 rates differ from those reported in other studies^9–11^, likely influenced by thorough and multiple pathological assessments. Nevertheless, the independently significant association between survival and R1 status in the PBD resection margin observed in the present study highlights the importance of separately assessing each resection margin and dissection plane in PCCA.

This study has several further limitations. Because the study included patients treated from 2010 to 2023, the results may have been influenced by changes in perioperative management, pathological assessment, and oncological strategies over time. For example, more patients in the R1 group were treated with adjuvant treatment, possibly affecting patient prognosis because adjuvant treatment reduced the negative association between R1 and survival. Additional analyses taking into account other possible confounders (that is, preoperative treatment, American Society of Anesthesiologists grade, PVE) revealed no change in outcomes. Because reassessment of patients was not feasible, particularly before 2018, the radicality status in these patients remained uncertain, which may have contributed to an overestimation of R0 resections^5,23^. Although only a positive PBD was negatively associated with survival in the present study, this does not necessarily imply that the other margins lack prognostic value, because positive margins were reported in only 1–18% of patients. The survival analyses included patients who died within 90 days of surgery, potentially affecting the assessment of oncological benefit of R0 *versus* R1. The main results remained consistent in a 90-day landmark sensitivity analysis.

Unlike previous studies that identified a negative impact of a positive radial margin on OS, this association was only relevant for longitudinal margin in the present study. Of the six standard resection margins and dissection planes, only a positive PBD resection margin was associated with worse prognosis, probably reflecting aggressive tumour biology. These discrepancies underscore the need for meta-analyses and future prospective large studies with standardized pathology protocols performed by expert HPB pathologists, with integration of molecular data, to better understand the prognostic and oncological significance of individual positive resection planes. Until then, oncological treatment strategies should be investigated in the setting of residual disease with individual resection margins and dissection planes considered.

## Supplementary Material

zraf160_Supplementary_Data

## Data Availability

Data will be made available upon reasonable request to the corresponding author. Requests should be accompanied by a statistical analysis plan.
