# Supplementary material for: Prognostic impact of individual resection and dissection margins in resected perihilar cholangiocarcinoma: retrospective study
Source: BJS Open. 2026 Feb 3;10(1):zraf160. doi: 10.1093/bjsopen/zraf160 (PMC12866661; doi:10.1093/bjsopen/zraf160)
Supplement: zraf160_Supplementary_Data [file zraf160_supplementary_data.docx]

**Prognostic Impact of Individual Resection and Dissection Margins in Resected Perihilar Cholangiocarcinoma**

Britte H. E. A. ten Haaft^1*^, Hasan A. Al-Saffar^2*^, Eva Roos^3^, Mahsoem Ali^1^, Heinz-Josef Klümpen^4^, Lynn Nooijen^5^, Lotte Franken^5^, Geert Kazemier^5^, Carlos Fernandez Moro^6^, Joanne Verheij^3^, Joris I. Erdmann^1#^, Christian Sturesson^2#^

^1^ Department of Surgery, Cancer Center Amsterdam, Amsterdam UMC, University of Amsterdam, Amsterdam, The Netherlands.

^2^ Division of Surgery and Oncology, Department of Clinical Science, Intervention and Technology, Karolinska Institute and Karolinska University Hospital, Stockholm, Sweden.

^3^ Department of Pathology, Cancer Center Amsterdam, Amsterdam UMC, University of Amsterdam, Amsterdam, The Netherlands.

^4^ Department of Medical Oncology, Cancer Center Amsterdam, Amsterdam UMC, University of Amsterdam, Amsterdam, The Netherlands.

^5^ Department of Surgery, Cancer Center Amsterdam, Amsterdam UMC, Vrije Universiteit, Amsterdam, The Netherlands.

^6^ Department of Clinical Pathology and Cancer Diagnostics, Karolinska University Hospital, Stockholm, Sweden

^*^ Shared first authorship

^#^ Shared senior authorship

**CORRESPONDING AUTHOR**

Joris I. Erdmann, MD PhD, Department of Surgery, Amsterdam UMC location University of Amsterdam, Cancer Center Amsterdam and Amsterdam Gastroenterology Endocrinology Metabolism, De Boelelaan 1117 (ZH-7F), 1081 HV Amsterdam

Email: [j.i.erdmann@amsterdamUMC.nl](mailto:j.i.erdmann@amsterdamUMC.nl)

**During review process:** Britte ten Haaft, MD | [b.tenhaaft@amsterdamUMC.nl](mailto:b.tenhaaft@amsterdamUMC.nl)

**Supplementary Materials - Index**

**Supplementary Methods**

**File 1. Overview of pathology assessment Page 3**

**File 2. All parameters assessed Page 6**

**File 3. Statistical analyses Page 7**

**Supplementary Figures and Tables**

**File 4. Supplementary Figure 1.** Joint association of R status

and N status with OS. **Page 9**

**File 5. Supplementary Figure 2.** Adjusted association of resection

planes with OS. **Page 10**

**File 6. Supplementary Figure 3.** Adjusted association of resection

planes with DFS. **Page 11**

**File 7. Supplementary Table 1.** Missing data **Page 12**

**File 8. Supplementary Table 2.** Additional pathology characteristics

including frozen sections according to R-status. **Page 14**

**File 9. Supplementary Table 3.** Sensitivity analysis without multiple

imputation and adjustment. **Page 15**

**Supplementary Methods**

#### File 1. Overview of pathology assessment (Translated from Dutch to English)

Hilar Lesion (Klatskin Tumor)

Fresh Specimen Handling

1. Measure (in three dimensions) and weigh the specimen; if present, describe and open the gallbladder.
2. Ink the liver parenchymal resection margin (if applicable) and, preferably in the fresh state, ink the perihilar soft tissue dissection margin. Do not ink areas covered by the peritoneum.
3. Inspect vascular structures and bile ducts. The surgeon typically marks both vessels and bile ducts, usually with beads. In case of uncertainty, consult the surgeon.
4. Transversely sample the hepatic duct margin (usually marked with a white bead) and ink the ‘non-true’ resection margin yellow after sampling (for orientation in microscopy).
5. Transversely sample the common bile duct (CBD) margin (usually marked with a green bead) and ink the ‘non-true’ resection margin green after sampling (for orientation in microscopy).
6.
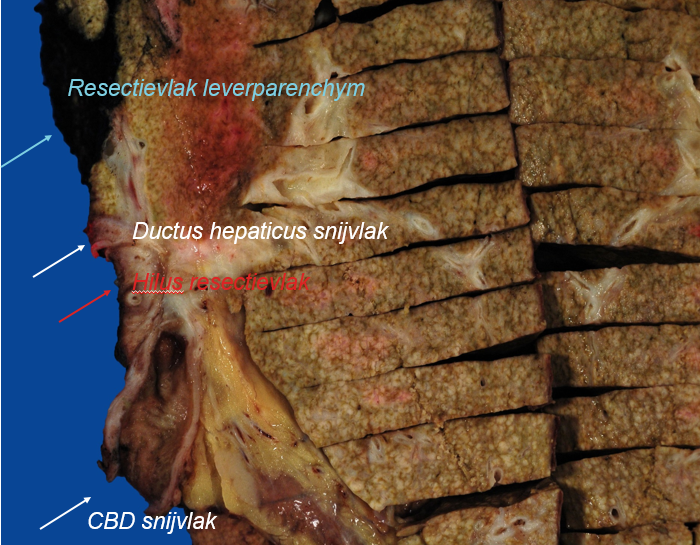
Sample the resection margins of the portal vein (usually marked with a blue bead) and hepatic artery (red bead).
7. Probe the bile ducts (along the length axis of the CBD-hepatic duct) and leave the probe in situ overnight in formalin for fixation. To facilitate fixation, incise the liver from the capsule towards the probe, keeping the lamellae intact and avoiding hilar cutting.
8. **After Fixation**
9. Incise the hilum along the probe in the CBD-hepatic duct.
10. Identify the tumor and measure its relationship to the hepatic duct (yellow ink) and the CBD (green ink).
11. Measure the minimal margin towards the liver parenchymal resection surface and the perihilar soft tissue dissection margin.
12. If applicable, document vascular invasion.
13. Further section the liver parallel to the first incision, ensuring minimal margins and other intrahepatic abnormalities are included when relevant.
14. Describe the liver tissue macroscopically (presence of steatosis, fibrosis, cirrhosis, etc.) and include relevant sections.

**Note: Low-threshold consultation with a supervisor is encouraged.**

**Report**

**Microscopy**

Liver Resection:

- Morphology of tumor
- Tumor boundaries: presence or absence of a capsule
- Resection margin status and minimal margin
- Possible angioinvasive growth
- Bile duct invasion
- Capsular breakthrough
- Possible tumor rupture, necrosis, and/or hemorrhage

Additionally, describe the results of immunohistochemistry and molecular analysis (if applicable). In the case of metastases, compare findings with the primary tumor.

Also, assess the surrounding liver tissue in terms of architecture, grading, and staging:

- Grading = degree of inflammatory activity
- Staging = degree of fibrosis

**Conclusion**

Liver Resection:

1. Nature and number of abnormalities, tumor type, differentiation grade, tumor size, perineural invasion, vascular invasion, resection margin status, minimal margin, and capsular breakthrough (if applicable).

**Note:**

- For hepatocellular carcinomas, define satellite nodules (≤ 2 cm in size and ≤ 2 cm from the tumor).
- For adenomas, specify the subtype.
- For Klatskin tumors, report all relevant margins and resection surfaces (liver parenchyma, hepatic duct, CBD, liver parenchyma, portal vein, and hepatic artery).

1. Additionally, report grading (degree of inflammatory activity) and staging (degree of fibrosis) in relation to the underlying disease of the surrounding liver tissue.
2. In post-chemotherapy cases, assess potential liver damage.
3. In cases of metastasis, compare findings with the primary tumor.

**File 2. All parameters assessed**

Clinicopathological parameters included patient demographics [age, sex, American Society of Anesthesiologists (ASA) score, body mass index (BMI), cirrhosis]; preoperative laboratory levels [P-bilirubin (μmol/L), P-albumin (g/L), Carbohydrate Antigen (CA) 19-9 (U/mL)] and radiological characteristics (BMC-type); preoperative interventions [endoscopic retrograde cholangiopancreatography (ERCP), percutaneous transhepatic cholangiography (PTC), and PVE], resection type, (neo)adjuvant treatments; postoperative complications [Clavien-Dindo grade ≥3 and 90-day mortality]; and pathological outcomes [tumor diameter (mm), tumor stage (TNM), differentiation grade, perineural (PN) growth, vessel involvement (VI), lymph nodes (LN – total count and ratio of positive lymph nodes (LN+))), frozen sections, resection and dissection planes, and the status of radical resection or residual disease (R0 or R1).

**File 3. Statistical analyses**

Categorical variables were reported as frequencies and percentages. Continuous variables were reported as medians with interquartile ranges (IQRs). Baseline characteristics for all patients were stratified according to R-status, and baseline differences were tested using Pearson’s chi-squared test for categorical data, and the Mann-Whitney U test for continuous data. Median follow-up was estimated using the reverse Kaplan-Meier method.

Multivariable Cox regression models were used to assess the association of R-status from different resection margins and dissection planes with OS and DFS, expressed as hazard ratios (HRs). These models included all resection margins and dissection planes and several potential confounders, including pre- and intraoperative factors (i.e., sex, age, ASA, CA19-9, lymphadenopathy [LN+], BMC type, type of resection, vascular reconstruction, preoperative systemic treatment, PVE, tumour diameter and differentiation grade). Continuous variables were modelled using restricted cubic splines to account for potential nonlinear covariate-outcome relationships and reduce the risk of residual confounding. The proportional hazards assumption was investigated using visual inspection of scaled Schoenfeld residuals and the Grambsch-Therneau test. Interactions were assessed using likelihood ratio tests in multivariable models, adjusted for the same factors as mentioned previously.

Flexible parametric Royston-Parmar survival models were used to show the association of a positive R-status with OS and DFS, for each resection margin and dissection plane.^17^ The models included the same covariates as the Cox regression model specified above, as well as time-varying coefficients if variables violated the proportional hazards assumption. Subsequently, regression standardization was used to estimate adjusted overall survival and the adjusted difference in OS over time for R0 vs R1, for each resection margin and dissection plane separately. A sensitivity analysis with a 90-day landmark was performed to evaluate long-term OS while excluding postoperative mortality.

Missing data were handled using flexible multiple imputation models (50 imputed datasets; 30 burn-in iterations) under the missing at random assumption. The imputation model was congenial with the analysis model, and included all covariates included in the analysis model, auxiliary variables (e.g., country and year of diagnosis), the event variable (i.e., death or censoring), and the Nelson-Aalen estimate of the cumulative baseline hazard. All effect estimates and standard errors were pooled across imputed datasets using Rubin’s rules.

A p-value of <0.05 was considered statistically significant. Statistical analysis was performed using R, version 4.4.1 (R Foundation for Statistical Computing), and Stata, version 18 (StataCorp), using the rms package and the stpm3 module^18,19^.

**Supplementary Figures and Tables**

**File 4. Supplementary Figure 1.** Joint association of R status and N status with OS.


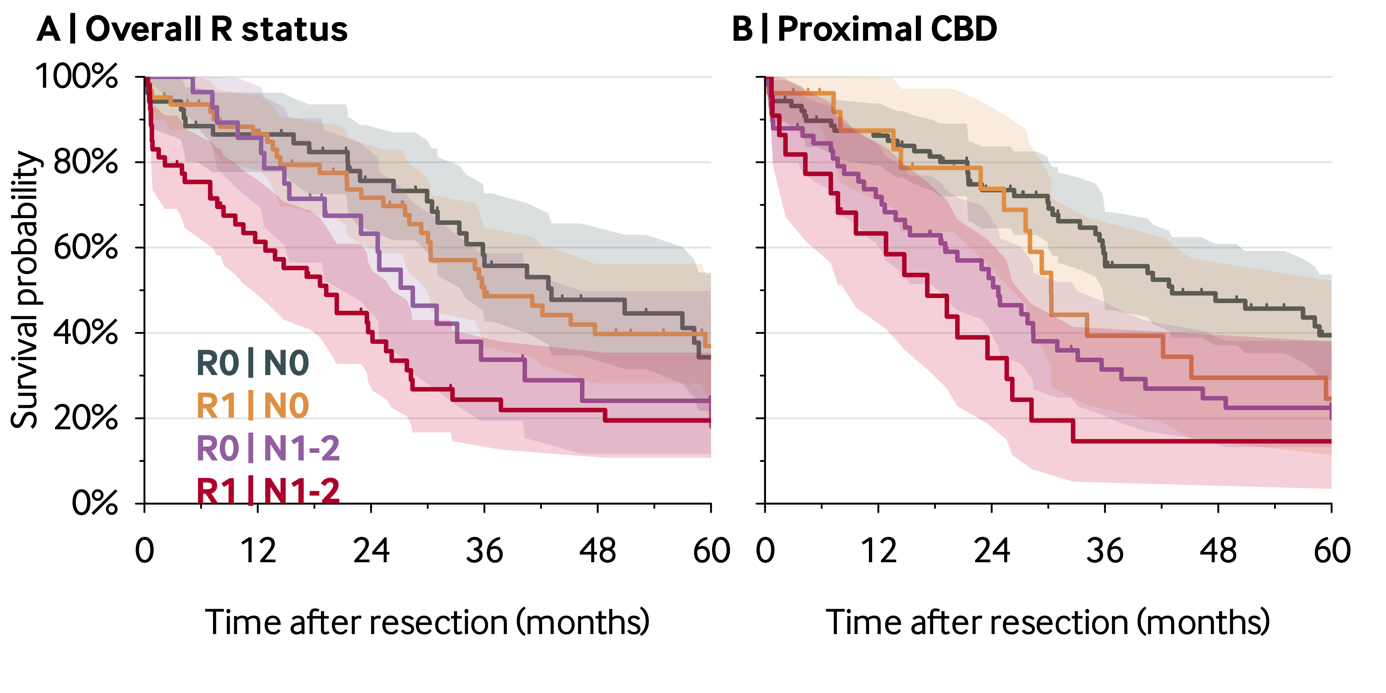


**File 5. Supplementary Figure 2. Adjusted association of resection planes with OS**. Adjusted OS was estimated using flexible parametric survival models that were adjusted for all variables included in Figure 2.





**File 6. Supplementary Figure 3. Adjusted association of resection planes with DFS**. Adjusted DFS was estimated using flexible parametric survival models that were adjusted for all variables included in Figure 2.





**File 7. Supplementary Table 1.** Number of missing data.

|  | Total (n=199) | | |
| --- | --- | --- | --- |
| Age, years | 2 | (1) |  |
| Sex, female | 0 | (0) |  |
| BMI, kg/m^2^ | 0 | (0) |  |
| ASA physical status | 0 | (0) |  |
| Bismuth Corlette | 0 | (0) |  |
| Prior abdominal surgery^1^ | 79 | (63) |  |
| Preoperative |  |  |  |
| Bilirubin (mmol/l) | 34 | (17) |  |
| Albumin (g/l) | 75 | (38) |  |
| Tumor marker (CA19.9) | 0 | (0) |  |
| Function (Amsterdam UMC) | 82 | (66) |  |
| Volume (Karolinska) | 20 | (28) |  |
| Neoadjuvant therapy | 7 | (4) |  |
| Biliary drainage | 0 | (0) |  |
| Portal vein embolization | 0 | (0) |  |
| Type of resection | 0 | (0) |  |
| Additional pancreatic resection | 0 | (0) |  |
| Vascular reconstruction | 20 | (10) |  |
| Postoperative complications |  |  |  |
| Clavien Dindo ≥3, <90 days | 7 | (4) |  |
| Mortality, <90 days | 0 | (0) |  |
| Adjuvant therapy | 8 | (4) |  |
| Tumor diameter, mm | 14 | (7) |  |
| Tumor stage^1^ – T status | 4 | (2) |  |
| Tumor stage^1^ – N status | 2 | (1) |  |
| Tumor stage^1^ – M status | 4 | (2) |  |
| Differentiation grade | 21 | (11) |  |
| Perineural invasion | 8 | (4) |  |
| Microscopic angioinvasion | 1 | (<1) |  |
| Liverparenchyma invasion^1^ | 0 | (0) |  |
| Distal bile duct frozen sections | 0 | (0) |  |
| Proximal bile duct frozen sections | 0 | (0) |  |
| Portal vein frozen sections | 0 | (0) |  |
| Hepatic artery frozen sections | 0 | (0) |  |
| Reresection performed | 0 | (0) |  |
| Residual disease |  |  |  |
| Distal (common bile duct) | 2 | (1) |  |
| Proximal (hepatic duct) | 1 | (<1) |  |
| Portal vein | 19 | (10) |  |
| Hepatic artery | 41 | (21) |  |
| Liver parenchyma | 11 | (6) |  |
| Periductal/circumferential | 23 | (12) |  |
| Missing resection margins and dissection planes^1^ | | |  |
| None (Complete) | 70 | (36) |  |
| 1 missing | 28 | (14) |  |
| 2 missing | 19 | (10) |  |
| 3 missing | 7 | (4) |  |
| 4 missing | 2 | (1) |  |
| Data are reported as n (%). ^1^ Only available for Amsterdam UMC | | | |

**File 8. Supplementary Table 2.** Additional pathology characteristics including frozen sections according to R-status.

|  | R0 (n=81) | | R1 (n=118) | | P value |
| --- | --- | --- | --- | --- | --- |
| Distal bile duct FS |  |  |  |  |  |
| Positive  Negative  NP | 6  63  12 | (7)  (78)  (15) | 10  88  20 | (9)  (75)  (17) | 0.874 |
| Proximal bile duct FS |  |  |  |  |  |
| Positive  Negative  NP | 13  42  26 | (16)  (52)  (32) | 22  49  47 | (19)  (42)  (40) | 0.352 |
| Portal vein FS |  |  |  |  |  |
| Positive  Negative  NP | 0  8  73 | (0)  (10)  (90) | 2  10  106 | (2)  (9)  (90) | 0.478 |
| Hepatic artery FS |  |  |  |  |  |
| Positive  Negative  NP | 0  4  77 | (0)  (5)  (95) | 1  6  111 | (1)  (5)  (94) | 0.707 |
| Reresection performed | 15 | (19) | 19 | (16) | 0.656 |
| Data are reported as n (%) or median (IQR). Variables with P<0.05 are shown in bold. FS: frozen section. | | | | | |

**File 9. Supplementary Table 3.** Sensitivity analysis without multiple imputation and adjustment.

|  | Hazard ratio for OS (95% CI) | | P value |
| --- | --- | --- | --- |
| Distal/common bile duct | 0.98 | (0.56 to 1.73) | 0.95 |
| Proximal bile duct | 1.62 | (1.13 to 2.33) | 0.011 |
| Portal vein | 1.02 | (0.59 to 1.76) | 0.94 |
| Hepatic artery | 0.90 | (0.29 to 2.83) | 0.85 |
| Liver parenchyma | 1.31 | (0.85 to 2.01) | 0.23 |
| Periductal/circumferential | 1.23 | (0.84 to 1.80) | 0.30 |
